# MRI correlates of cognitive improvement after home-based EEG neurofeedback training in patients with multiple sclerosis: a pilot study

**DOI:** 10.1007/s00415-021-10530-9

**Published:** 2021-03-30

**Authors:** Daniela Pinter, Silvia Erika Kober, Viktoria Fruhwirth, Lisa Berger, Anna Damulina, Michael Khalil, Christa Neuper, Guilherme Wood, Christian Enzinger

**Affiliations:** 1 Department of Neurology, Medical University of Graz, Auenbruggerplatz 22, Graz, Austria; 2 Research Unit for Neuronal Plasticity and Repair, Medical University of Graz, Graz, Austria; 3 BioTechMed-Graz, Graz, Austria; 4 Institute of Psychology, University of Graz, Graz, Austria; 5 Laboratory of Brain-Computer Interfaces, Institute of Neural Engineering, Graz University of Technology, Graz, Austria; 6 Division of Neuroradiology, Vascular and Interventional Radiology, Department of Radiology, Medical University of Graz, Graz, Austria

**Keywords:** Cognitive training, Multiple sclerosis, Neurofeedback, DTI, Resting-state fMRI

## Abstract

**Objective:**

Neurofeedback training may improve cognitive function in patients with neurological disorders. However, the underlying cerebral mechanisms of such improvements are poorly understood. Therefore, we aimed to investigate MRI correlates of cognitive improvement after EEG-based neurofeedback training in patients with MS (pwMS).

**Methods:**

Fourteen pwMS underwent ten neurofeedback training sessions within 3–4 weeks at home using a tele-rehabilitation system. Half of the pwMS (*N* = 7, responders) learned to self-regulate sensorimotor rhythm (SMR, 12–15 Hz) by visual feedback and improved cognitively after training, whereas the remainder (non-responders, *n* = 7) did not. Diffusion-tensor imaging and resting-state fMRI of the brain was performed before and after training. We analyzed fractional anisotropy (FA) and functional connectivity (FC) of the default-mode, sensorimotor (SMN) and salience network (SAL).

**Results:**

At baseline, responders and non-responders were comparable regarding sex, age, education, disease duration, physical and cognitive impairment, and MRI parameters. After training, compared to non-responders, responders showed increased FA and FC within the SAL and SMN. Cognitive improvement correlated with increased FC in SAL and a correlation trend with increased FA was observed.

**Conclusions:**

This exploratory study suggests that successful neurofeedback training may not only lead to cognitive improvement, but also to increases in brain microstructure and functional connectivity.

## Introduction

Cognitive impairment (CI) is present in 40–70% of patients with MS (pwMS) and now viewed as one of the most disabling symptoms [1, 2]. While some experts concluded that the efficacy of traditional cognitive rehabilitation in pwMS is low, inconclusive or preliminary [3–5], others highlighted that the evidence for beneficial effects of cognitive rehabilitation has become increasingly more convincing [6, 7]. Recently, there has been a call for cognitive interventions based on and guided by findings from neuroimaging studies to improve validation [8].

Home-based EEG neurofeedback (NF) therefore represents a promising tool for cognitive rehabilitation in MS, because for pwMS attendance of in-person cognitive rehabilitation is frequently challenging due to high demands of family and profession [7, 9]. In NF applications, brain activity is recorded and fed back to the users in real-time. This enables voluntary modulation of one’s own brain activity, which might lead to cognitive, motor, affective, or behavioral improvements [10].

Notwithstanding these aspects, to date, only a few studies have investigated the efficacy of NF training on cognition, depression and fatigue in MS so far [8, 11, 12].

Improved cognitive function after NF focusing on upregulation of the sensorimotor rhythm (SMR, 12–15 Hz) in the EEG has been reported in healthy individuals and in patients with stroke, epilepsy and MS [10, 12–15]. An increase in SMR activity is associated with a reduction or inhibition of sensorimotor interferences, which might disturb cognitive processing [12, 16, 17]. However, the underlying (putatively neuroplastic) mechanisms of successful SMR NF training still remain unclear, although it is assumed that the cortico-basal ganglia-thalamo-cortical loop (CBGTC) represents a target, given its involvement in sensorimotor, cognitive, and associative functions [16, 18].

In a recent pilot study, we found that pwMS who successfully learned to self-regulate sensorimotor rhythm (SMR, 12–15 Hz) also showed cognitive improvements after training [12]. We here aim to specifically investigate MRI correlates of such cognitive improvement after EEG-based NF training in pwMS.

## Methods

### Patients

For this pilot study, we analyzed comprehensive brain MRI data of fourteen patients with relapsing–remitting (RRMS) or secondary progressive (SPMS) MS (Table 1), which are described in more detail in our previous publication [12].All patients performed the same NF training and pre-post assessment. Post-hoc, patients were divided into two groups (responders vs non-responders, *N* = 7 each) depending on their changes in cognitive performance as assessed with the Brief Repeatable Battery of Neuropsychological Tests (BRB-N) [19]. For statistical analysis of cognitive data, T-normative scores of the single BRB-N sub-tests were used. The global T-score was performed using the composite score (mean) of all single subscores. Before training, the groups were comparable in their cognitive performance (all *p* > 0.08) [12]. Intra-individual comparisons of the global T-score assessed during pre- and post-assessment were performed by using critical difference analysis. The critical difference was established using the test–retest reliability of the test and its standard deviation. The global cognition critical T-score difference value was 4.92 [12]. Patients with significant improvements in global cognition (post–pre difference in overall T-scores), were defined as “responders” [12]. “Non-responders” did not demonstrate significant changes in cognitive function when comparing the pre- and post-assessments. The binominal probability (*p* = 0.0002) showed that the number of observed significant performance improvements in the overall BRB-N score (*N* = 7) was higher than the chance level [12].

**Table 1:** Patient characteristics for baseline assessment (pre NF training)

	Responders (*N* = 7)	Non-Responders (*N* = 7)	*p*
Sex, female, %	57.1	42.9	0.83
Age, years	36.9 (4.2)	41.0 (1.6)	0.83
Education, years	15.4 (1.3)	14.4 (1.4)	0.83
EDSS	3.0 (3.5)	2.0 (3.5)	0.83
DD, years	13.4 (3.0)	7.2 (1.9)	0.83
RRMS, %	85.7	100	0.83
Cognition (BRB-N)			
BL total score	44.4 (4.2)	48.1 (3.6)	0.96
Post–pre difference of total cognitive score	10.8 (3.0)	− 1.5 (3.5)	0.02
MRI measures			
NBV cm^3^	1437.71 (59.62)	1476.38 (92.07)	0.83
CGM cm^3^	592.06 (41.76)	601.88 (61.70)	0.83
T2-LL cm^3^	19.3 (22.5)	11.3 (23.1)	0.83
TVol cm^3^	14.37 (2.96)	14.90 (1.57)	0.93
HVol cm^3^	7.09 (1.27)	7.70 (0.57)	0.83
CVol cm^3^	6.60 (1.45)	7.11 (1.02)	0.83
PuVol cm^3^	9.20 (1.77)	9.91 (1.71)	0.83
PaVol cm^3^	3.48 (0.65)	3.50 (0.48)	0.83

Nominal data is presented in % (Chi-Square test). For all other variables, Median and IQR are presented (MWU). FDR-adjusted p-values are presented

*BL *baseline (pre-training), *CGM *cortical grey matter, *CVol *nucleus caudatus volume, *DD *disease duration, *EDSS *Expanded Disability Status Scale, *HVol *hippocampal volume, *NBV *normalized brain volume, *NF *neurofeedback, *PaVol *Pallidum volume, *PuVol *Putamen volume, *RRMS *relapsing–remitting MS, *T2-LL *T2—lesion load, *TVol *thalamic volume

This study was approved by the ethics committee of the Medical University of Graz (27–520 ex 14/15). All participants gave written informed consent. The present study is in accordance with the Declaration of Helsinki.

### Neurofeedback training and EEG data analysis

All patients performed 10 NF training sessions within 3–4 weeks at their home using a tele-rehabilitation system. In short, patients were trained using an EEG headset, portable 10-channel EEG amplifier (NeXus-10 MKII, Mind Media B.V.) and laptop, while the therapist (SK) was able to remotely monitor NF training and EEG data quality. Before the first NF training session, the therapist prepared, instructed, and trained the patients in how to use the NF system at the patients’ home. During NF training, patients received visual feedback of their own SMR power (12–15 Hz), theta power (4–7 Hz), and beta power (21–35 Hz). The goal was to increase SMR power above and to keep theta and beta power below these thresholds. One NF training session comprised one baseline run (3 min: relaxation) and six feedback runs (each 3 min), targeting a physically relaxed and mentally focused state [12]. EEG data analyses were performed as described previously [12].

### MRI protocol

MRI was performed on a 3 T Tim-Trio scanner (Siemens Healthcare, Erlangen, Germany). Structural 3D images with high resolution were acquired by means of a T1-weighted MPRAGE sequence with 1 mm isotropic resolution (TR = 1900 ms, TE = 2.19 ms, 176 slices). A T2-weighted fluid-attenuated inversion recovery (FLAIR) sequence with 1 × 1 × 3 mm resolution served for the assessment of the hyperintense T2-lesion load (T2-LL) (TR = 9000 ms; TE = 69 ms, 44 slices).

Diffusion tensor imaging (DTI) was acquired with 2 × 2 × 2 mm resolution (TR = 3321 ms; TE = 75 ms, b value = 1,000 s mm^2^, 64 directions). Resting-state fMRI (rfMRI) data was acquired with 2 × 2 × 2 mm (TR = 1000, TE = 35, 300 volumes). Participants were asked to close their eyes during rfMRI. The total imaging time was approximately 20 min.

### Structural MRI analyses

The burden of focal white matter inflammation (respective residual footprints of it) was estimated by the T2-lesion load (T2-LL). Lesion identification was performed by a single experienced rater (AD). Afterwards, a semi-automated lesion growing algorithm was used to assess T2-LL [20]. After lesion filling with the FSL lesion filling toolbox, normalized brain volume (NBV) and cortical grey matter (CGM) brain volumes were assessed from baseline T1-weighted MPRAGE images using SIENAX and deep grey matter volumes (thalamus (TVol), caudate nucleus (CVol), putamen (PuVol), pallidum (PaVol) and hippocampus (HVol)) were assessed using FIRST, both part of the FMRIB Software Library (FSL).

### Microstructural (DTI) analyses

Diffusion tensor imaging analysis was performed using FDT (fMRIB’s Diffusion Toolbox) and TBSS (Tract-Based Spatial Statistics, both part of fMRIB’s Software Library 5.0.9).

Raw images were pre-processed using Eddy Current correction. A brain mask was created using BET (Brain Extraction Tool). Maps for fractional anisotropy (FA) were generated. Subsequently, individual difference maps of FA (FA post-NF training—FA pre-NF training) were created for each subject (deltaFA). Voxel-wise statistical analysis of FA data and delta FA was carried out using TBSS.

The FA skeleton was thresholded at 0.20 to include major white matter pathways but avoid peripheral tracts (vulnerable to inter-subject variability). Each subject’s FA map was then projected onto the mean skeleton. Voxel-wise cross-subject statistics (threshold-free cluster enhancement) was applied. We used non-parametric testing as implemented in ‘‘FSL randomise’’ (5000 permutations) for calculating group contrasts, potential differences in FA increases or decreases between two groups (using delta FA) and voxel-wise correlations with behavioral measures (cognitive improvement). The anatomical location of significant clusters was determined by reference to the fiber tract-based atlas of human white matter (JHU ICBM-DTI-81 White-Matter Labels, JHU White-Matter Tractography Atlas, Juelich Histological Atlas), as implemented in FSL.

### Functional (rfMRI) analyses

In the first step, individual resting-state data were preprocessed using FEAT (FMRIB’s Expert Analysis Tool). Individual pre-statistical processing included: motion correction using MCFLIRT, brain extraction, spatial smoothing, and registration. Next, ICA-based automatic removal of motion artifacts (ICA AROMA) was performed [21]. Denoised data was then registered to the standard MNI152 template. “Multivariate Exploratory Linear Optimized Decomposition into Independent Components” (MELODIC) was used to decompose data of all 28 rfMRI scans (i.e., of the 14 patients before and after NF training) into 15 independent components. We focused on three networks: the default mode network (DMN), the sensorimotor network (SMN), and the salience network (SAL), which were objectified by an overlap of the network identified by MELODIC with the Smith 10 templates [22]. The set of spatial maps from the group-average analysis was used to generate subject-specific versions of the spatial maps, and associated timeseries, using dual regression [23]. First, for each subject, the group-average set of spatial maps is regressed (as spatial regressors in a multiple regression) into the subject's 4D space–time dataset. This results in a set of subject-specific timeseries, one per group-level spatial map. Next, those timeseries are regressed (as temporal regressors, again in a multiple regression) into the same 4D dataset, resulting in a set of subject-specific spatial maps, one per group-level spatial map. We then tested for group differences, potential increases or decreases (using individual delta rfMRI maps for each network) and correlations with cognitive data and SMR increase using “FSL Randomise (5000 permutations)”. Significant regions were identified using the Harvard–Oxford cortical and subcortical atlas implemented in FSL.

### Statistical analyses

Demographic, clinical, and MRI data were analyzed with the Statistical Package of Social Science (IBM SPSS Statistics 26). The level of significance was set at 0.05. FDR-adjustment of *p* values for multiple comparison correction was used where applicable. Mann–Whitney *U* Test was used to compare groups and Spearman correlations were performed to explore associations between MRI variables and cognitive improvement or SMR increase. Cognitive improvement was defined by post vs pre-training increase of overall cognitive function assessed by the BRB-N. SMR increase was assessed by the average difference in SMR power between the last NF run and the baseline run within the NF training sessions.

## Results

**Fig. 1:**
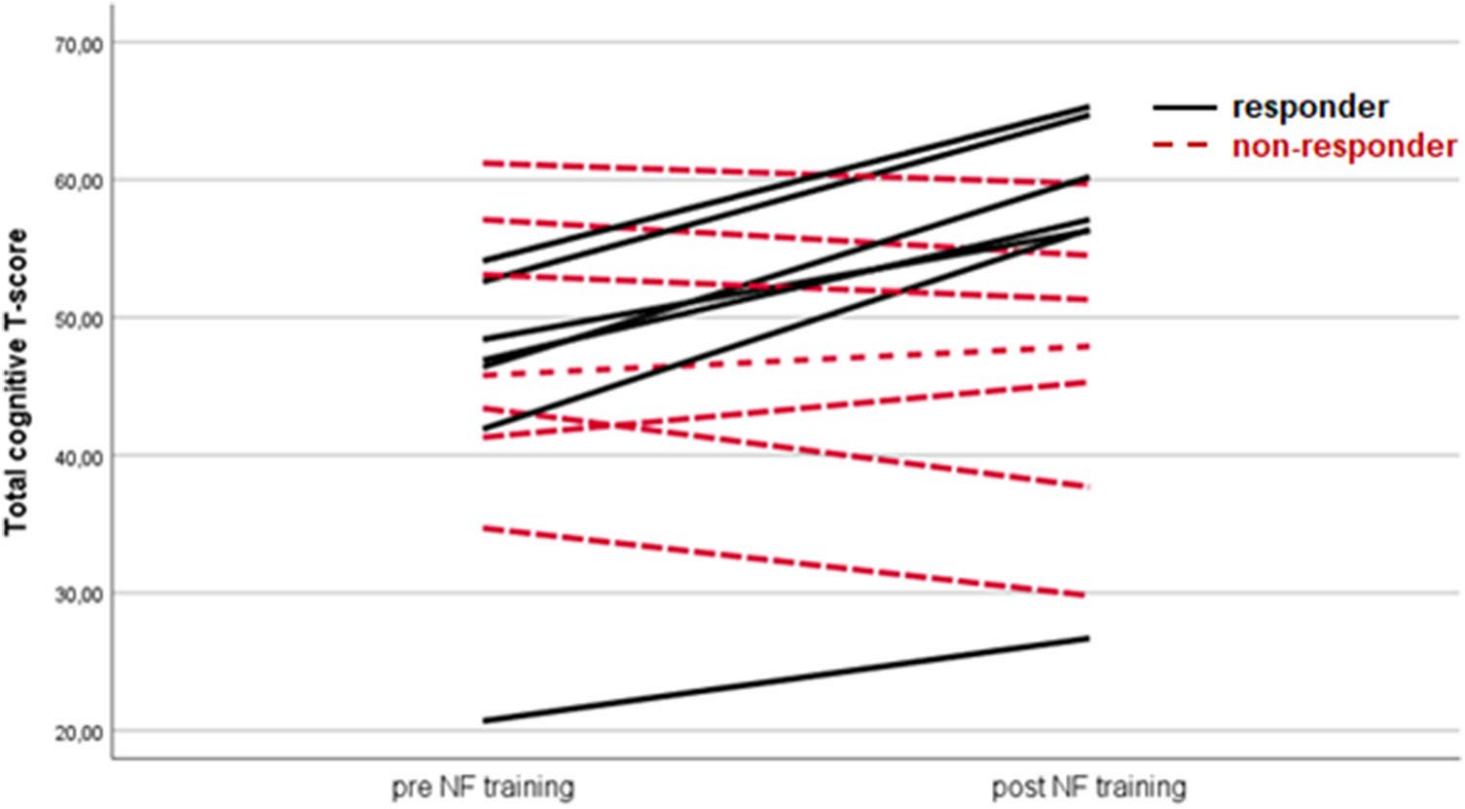
Individual change of the overall cognitive T-score for responders (black line) and non-responders (red dotted line)

“Responders” where defined by significant improvements in global cognition (post–pre difference in overall T-scores), and found to be also able to self-regulate SMR. “Non-responders” did neither demonstrate any changes in cognitive function nor were they able to modulate their own brain activity, when comparing the pre- and post-assessments (Fig. 1).

### Comparison of baseline structural MRI parameters between groups

The amount of focal MS-related white matter damage (hyperintense T2-lesion load; T2-LL) did not differ between responders and non-responders at baseline (pre-NF training), neither did baseline cortical grey matter volume (CGM) and normalized brain volume (NBV). Subcortical volumes of the thalamus, caudate nucleus, pallidum, putamen, and hippocampus did also not differ significantly between these groups at baseline (Table 1).

We observed no significant correlations across the entire cohort between baseline morphological MRI parameters and cognitive improvement (*p* > 0.392) or SMR improvement (*p* > 0.095).

### Comparison of white matter integrity changes between groups

**Fig. 2:**
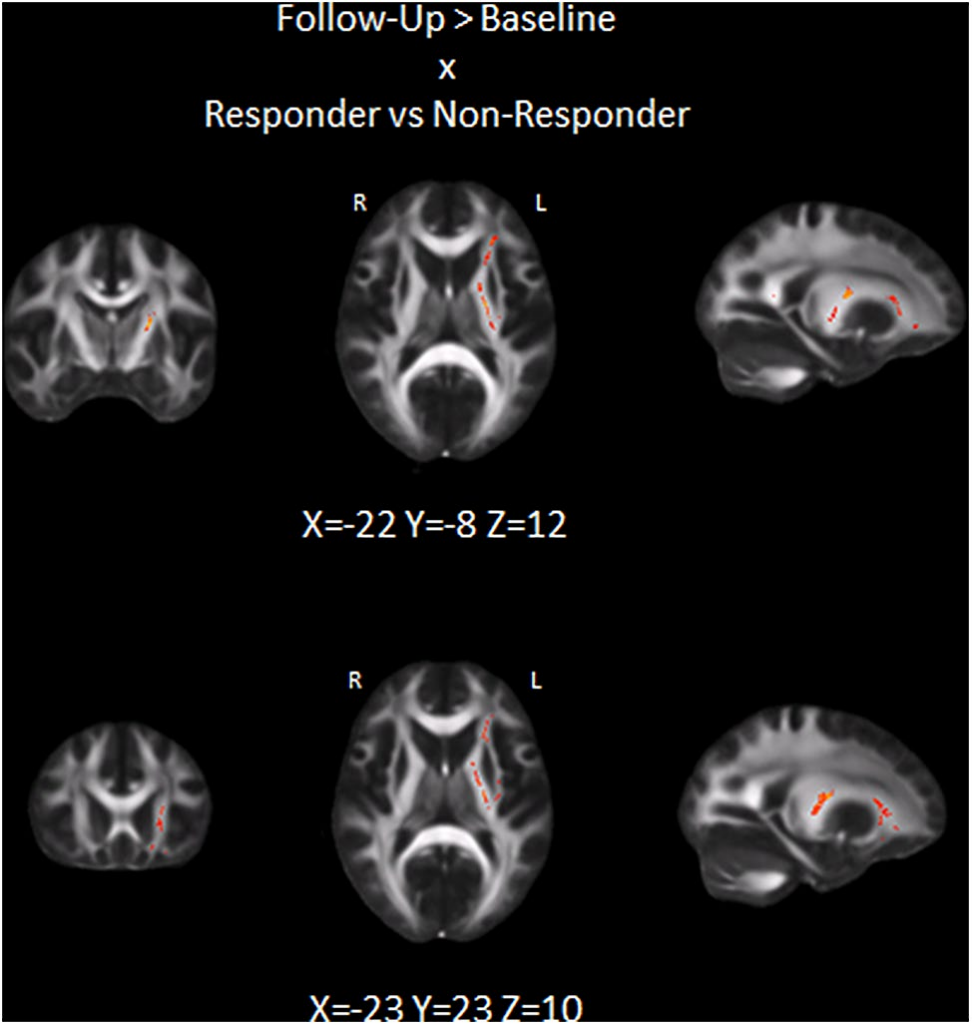
Interaction effect showing increased FA (post > pre NF training) in responders compared to non-responders in the corticospinal tract and anterior thalamic radiation (*p* < 0.05)

Measures of microstructural white matter integrity (FA) did not differ between both groups at baseline. A significant interaction effect (between-group comparison of individual delta FA maps (post–pre NF-training FA)) showed increased FA in responders compared to non-responders in the left corticospinal tract, left anterior thalamic radiation, left inferior longitudinal fasciculus, right forceps major, right optic radiation and left inferior fronto-occipital fasciculus after NF-training (Fig. 2).

### Changes in white matter integrity associated with cognitive improvement or SMR increase

**Fig. 3:**
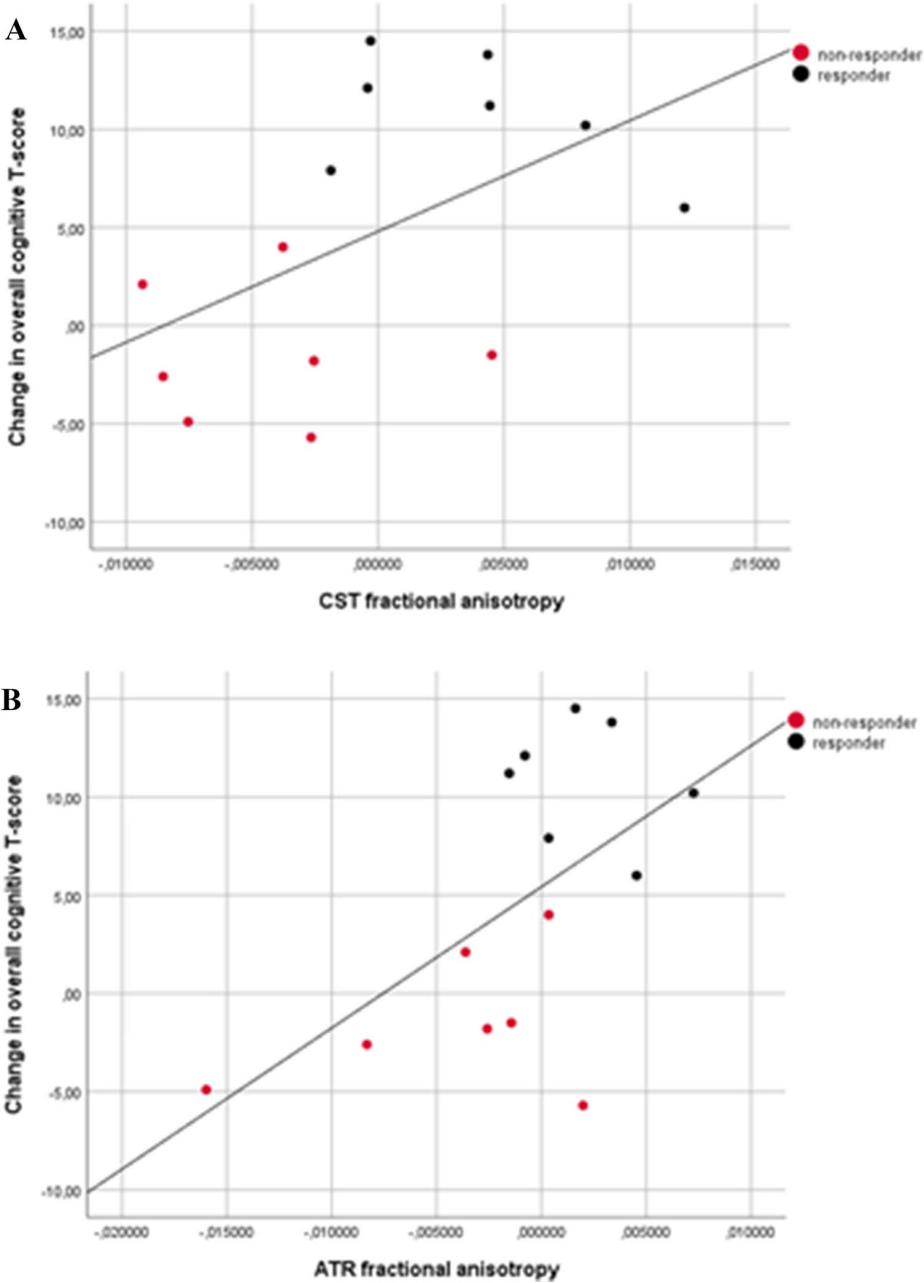
Scatterplot for associations between change in overall cognitive T-score and change in extracted mean fractional anisotropy of the **a** corticospinal tract (CST) and **b** anterior thalamic radiation (ATR). Responders (black) and non-responders (red)

Across the entire group, voxel-wise analysis revealed a trend for the positive correlation between increased FA and improved overall cognitive function in the left CST and ATR (*p* = 0.08; Fig. 3).

Voxel-wise analyses revealed no significant associations between changes in white matter integrity and increases in SMR power during NF training.

### Comparison of functional connectivity between groups

**Fig. 4:**
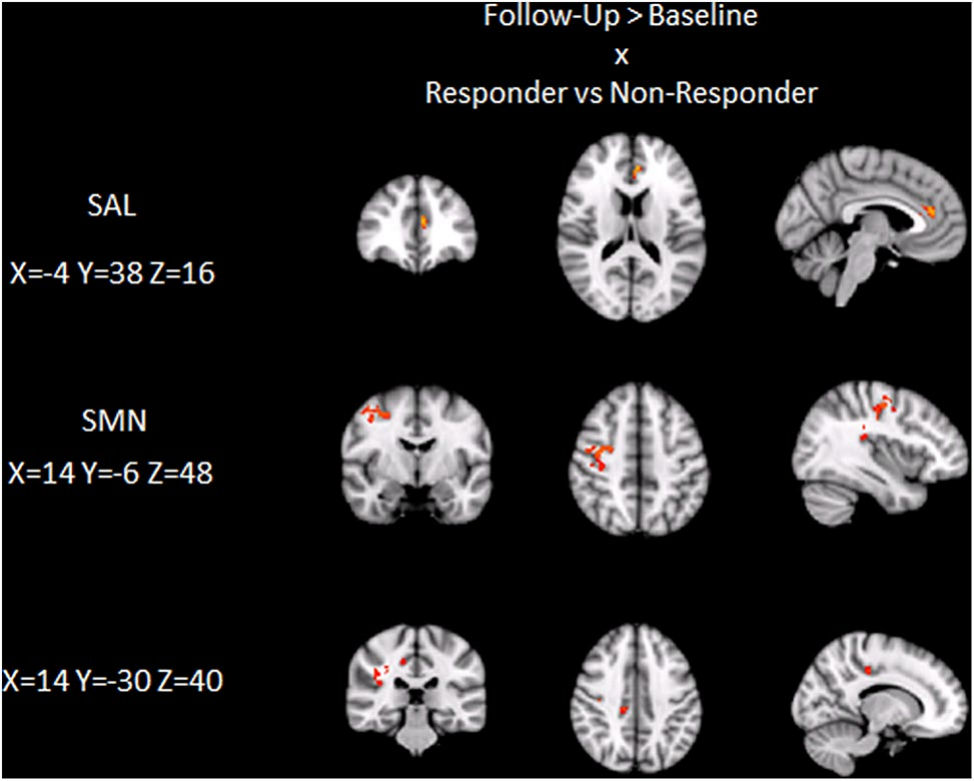
Interaction effect showing increased FC (post > pre NF training) in the salience network (SAL) and sensorimotor network (SMN) in responders compared to non-responders (*p* < 0.05)

**Fig. 5:**
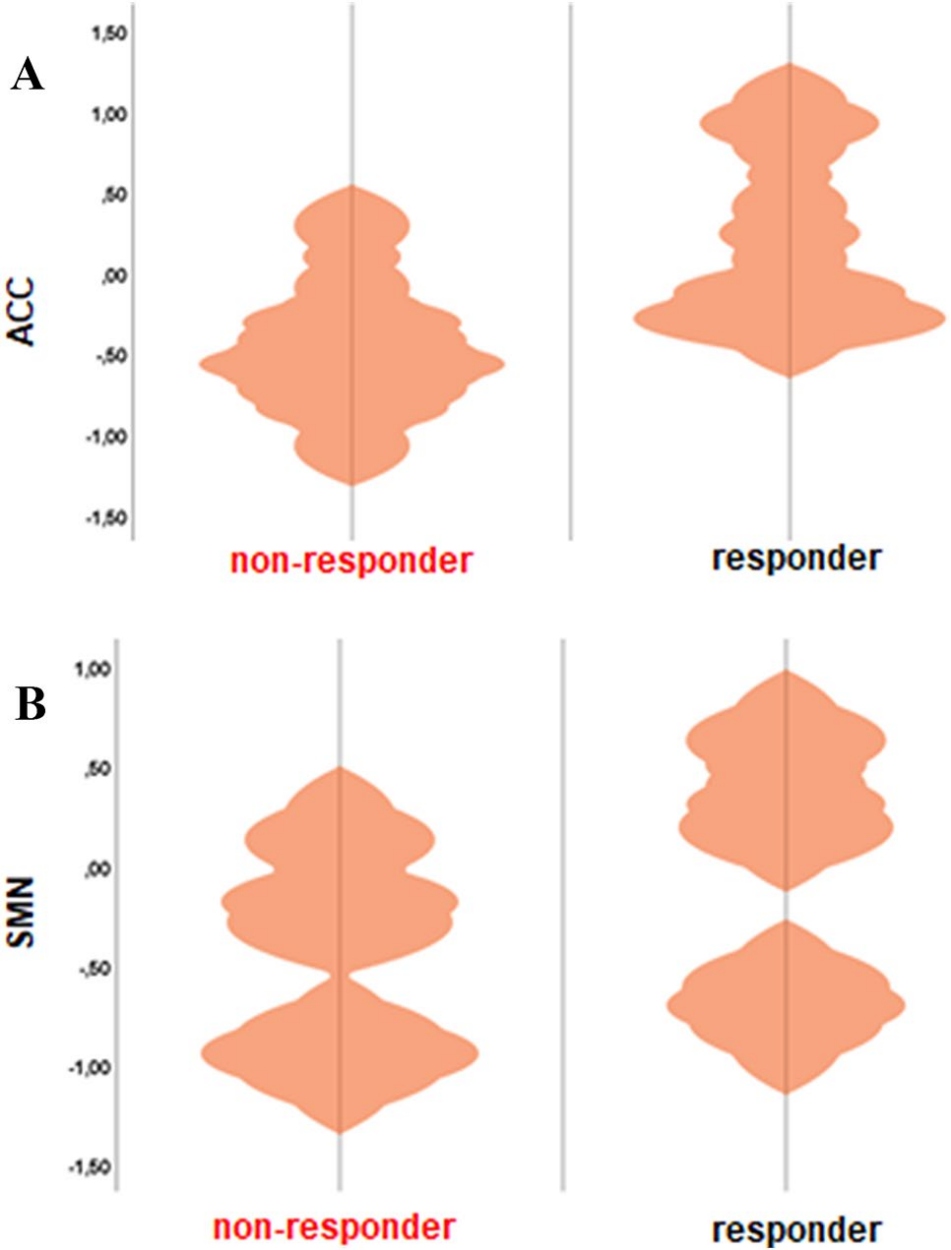
Violin plots showing changes in FC (post > pre NF training) in the salience network (SAL) and sensorimotor network (SMN) in responders compared to non-responders

Functional connectivity (FC) in the DMN, SAL and SMN did not differ between groups at baseline. Between-group comparison of individual delta rfMRI maps showed no significant differences of change in FC in the DMN between responders compared to non-responders. A significant interaction effect revealed increased FC in responders compared to non-responders after NF training in the SAL (anterior cingulate cortex, ACC; *p* < 0.05) and SMN (precentral gyrus, posterior cingulate gyrus; *p* < 0.05; Figs. 4 and 5).

### Changes in functional connectivity associated with cognitive improvement and SMR change

**Fig. 6:**
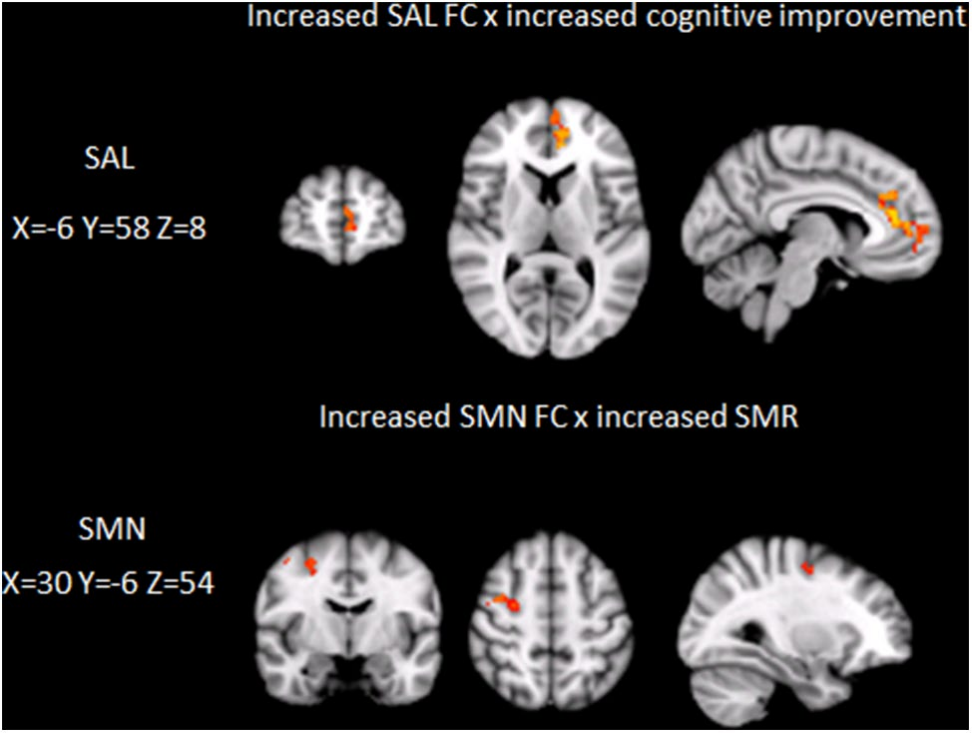
Increased FC in the salience network (SAL) associated with cognitive improvement across the entire cohort. Increased FC of the sensorimotor network (SMN) associated with increase of SMR power across the entire cohort (*p* < 0.05)

Voxel-wise analyses revealed increased FC associated with improved cognitive function in the SAL (ACC; Fig. 6) across the entire group.

Voxel-wise analyses showed that increased FC of the SMN (Fig. 6) was related to higher SMR increase.

### Association between changes in white matter integrity and changes in functional connectivity

Higher extracted FA increases in the CST peak voxel correlated with increased FC of the ACC peak voxel (*r* = 0.666; *p* = 0.009) and thalamus (*r* = 0.556; *p* = 0.039) and increased FA of the ATR peak voxel was associated with increased FC of the ACC (*r* = 0.385; *p* = 0.046) across the entire cohort.

## Discussion

This exploratory study revealed increases in white matter integrity (FA) and functional connectivity (FC) associated with cognitive improvement after home-based neurofeedback training (NF) in patients with multiple sclerosis (pwMS).

Previous studies suggested that voluntary NF strengthening of neural networks is governed by a reward-modulated Hebbian learning rule [24]. Knowing that neurons that “fire together wire together” essentially helped to better understand mechanisms of learning and brain plasticity [25]. In line with the few previous DTI studies investigating NF-related white matter changes [26–28], we found increased white matter integrity in NF responders. While Ghaziri et al. [28] were the first to demonstrate increased FA in white matter pathways implicated in attention after a NF-attention training, another study reported NF-specific increases in white matter integrity within the SMN after motor imagery NF training [26]. Both studies investigated healthy individuals, but increases in thalamo-cortical FA were also observed in two young moderately affected traumatic-brain injury patients after a cognitive NF-training [27]. Our findings of increased FA in the corticospinal tract (CST) and anterior thalamic radiation (ATR) in pwMS comply with our observation of FC changes and current theoretical assumptions of NF-related neuroplasticity. Therefore, while increased CST integrity may likely occur specifically after SMR NF, the ATR seems to be crucial for NF in general (irrespective of the NF target) [24, 29].

Three interactive stages and brain regions have been suggested to be crucial for successful NF training [24]. First, the early period of learning is dominated by frontal brain regions, supported by the striatum, generating different representations (e.g., increasing SMR) and maintaining those that produce a positive feedback signal (e.g., visual feedback). Secondly, the winning frontal representations are activated and modify connections to and within the thalamus. Thirdly, the target brain state along with subjective experiential representations acts as secondary reinforcer for closing the interoceptive homeostatic loop, processed by the anterior insular region [24].

The ATR is connecting the dorsolateral prefrontal cortex (involved in executive functions, working memory and motor regulation) and anterior cingulate cortex (ACC; involved in attention, reward anticipation and decision-making) [29, 30] to the thalamus. This structural connection also appears to be crucial for the functional salience network (SAL), also called executive-control network [22]. SAL core regions such as the ACC (stage 1), thalamus (stage 2) and anterior insula (stage 3) play a general role in NF trainings and central role in cognitive control and the recruitment of appropriate functional brain-behavior networks modulating behavior induced by a cortico-striato-thalamo-cortical loop [31]. Interestingly, we observed associations between FC of the SAL with cognitive improvement and SMR increase, whereas FC of basal ganglia (BG) were only related to SMR increase. BG are involved in learning, visuomotor integration, and motivational processing [29]. Notably, our findings are consistent with a meta-analyses of real-time fMRI NF studies showing that brain self-regulation involves a complex regulation network, including the anterior insula, ACC and basal ganglia, independent of the applied protocol or target region of interest [29].

The symbiotic interplay between structure and function is at the heart of NF training [25]. By targeting functional changes, one can induce changes in the brain’s structural architecture, which would in turn support a more persistent functional reorganization [25]. The observed association between microstructural (increased FA in the left ATR) and functional changes (increased FC of the ACC) after successful NF training might reflect this assumption.

Compared to other forms of cognitive rehabilitation, the advantage of self-administered EEG NF is twofold. First, it allows the subject to find his or her own cognitive task associated with positive reinforcement and thus to maximize the effect on the neurophysiological characteristics targeted by the NF protocol. Secondly, home-based NF training provides high flexibility to incorporate training into everyday life [30, 32]. Despite limited data in MS, home-based NF training therefore may be considered as a promising technique for cognitive neurorehabilitation.

Some limitations have to be considered when interpreting our results. First, unfortunately due to the low sample size, we were not able to explore group-specific associations between MRI changes and cognitive improvement. However, observing significant differences in such small groups underlines the high sensitivity of DTI and resting-state fMRI to explore training-related changes, if confirmed by other studies in different samples. Furthermore, the lack of a control group limits interpretability of our findings. While increased FA is commonly interpreted to reflect more efficient axonal signal conduction, increases in FC have been controversially discussed in patients with MS. In our sample, positive correlations between improved cognition and FC suggest beneficial effects of increased FC. Secondly, consistent with prior studies, only half of our investigated patients were able to successfully increase SMR. Given the comparability of both groups regarding demographics and brain morphology it still remains to be elucidated why some people are not able to voluntarily control their brain oscillations. Interestingly, also in healthy cohorts, some participants fail to attain self-regulation [29]. Future research should seek to examine further potentially influential variables to differentiate between responders and non-responders prior to NF training. Thirdly, stability of cognitive improvement and transfer to everyday tasks remains to be explored in larger studies, including follow-up over longer time periods.

To conclude, our exploratory study suggests that successful SMR NF training may not only lead to cognitive improvement, but also to increased white matter integrity and FC in brain regions associated with self-regulation, motor, and cognitive function. Therefore, NF seems to be a promising tool that deserves to be further explored for cognitive rehabilitation in pwMS.

## Data Availability

Data that support the findings of this study are available from the corresponding author upon reasonable request.
